# Access to child-appropriate medicines: an exploratory survey of the use of paediatric use marketing authorisation products in the UK

**DOI:** 10.1007/s00431-025-05987-z

**Published:** 2025-01-25

**Authors:** Mandy Wan, Amin Houshian, Stephen Tomlin, Asia N. Rashed

**Affiliations:** 1 Pharmacy Department, Guy’s and St Thomas’ NHS Foundation Trust, Evelina London Children’s Hospital, Westminster Bridge Road, London, SE1 7EH UK; 2 Institute of Pharmaceutical Science, King’s College London, London, UK; 3 Pharmacy Department, King’s College Hospital, London, UK; 4 Pharmacy Department, Great Ormond Street Hospital for Children, London, UK

**Keywords:** Access, Access to medicine, Accessibility, Medicines, Off-label use, Unlicensed use, Paediatric medicines

## Abstract

The Paediatric Use Marketing Authorisation (PUMA) was introduced in the European Union to incentivise the development of off-patent medicines in children. However, there is limited data on the accessibility of PUMA products at the healthcare provider level. This study aimed to identify factors affecting real-world accessibility to PUMA products in the United Kingdom (UK). Inductive thematic analyses of the archives of the Neonatal and Paediatric Pharmacy Group (NPPG) online forum were conducted. A web-based survey was also distributed to NPPG members in September 2022 regarding the availability of PUMA products in their organisations. Thematic analysis generated five themes: authorisation, availability, affordability, appropriateness and acceptability. Restricted scope of the product’s marketing authorisation, market access variation, higher cost of PUMA products, product appropriateness and patient acceptability were reasons for continued off-label use and use of unlicensed products in clinical practice. *Conclusion*: Despite targeted legislative efforts to bring off-label uses in children into authorised use, this study provides evidence that authorisation alone does not equate to market availability, which in turn does not guarantee patient access. The study findings also suggest that cost pressure drives local procurement decisions and overshadows the long-standing problems associated with off-label use and manipulation of medicines in children.

**Table:** 

**What is already known about this subject** • *The Paediatric Use Marketing Authorisation (PUMA) was introduced in the European Union (applied to the UK at the time) to incentivise the development of off-patient medicines exclusively for use in the paediatric population.* • *It is widely acknowledged that the PUMA concept has not achieved its intended goal, as evidenced by the few products authorised through this route.*
**What this study adds** • *This study shows inequalities in children's access to PUMA products in the UK.* • *Determinants impeding patient access to child-appropriate paediatric medicines can be categorised into five dimensions: authorisation, availability, affordability, appropriateness, and acceptability.*

**What is already known about this subject**

• *The Paediatric Use Marketing Authorisation (PUMA) was introduced in the European Union (applied to the UK at the time) to incentivise the development of off-patient medicines exclusively for use in the paediatric population.*

• *It is widely acknowledged that the PUMA concept has not achieved its intended goal, as evidenced by the few products authorised through this route.*

**What this study adds**

• *This study shows inequalities in children's access to PUMA products in the UK.*

• *Determinants impeding patient access to child-appropriate paediatric medicines can be categorised into five dimensions: authorisation, availability, affordability, appropriateness, and acceptability.*

## Introduction

In recent decades, regulatory frameworks have been established to increase the availability of medicines authorised for children [1]. The Paediatric Use Marketing Authorisation (PUMA) was introduced in the European Union (EU) in 2007, as part of the European Paediatric Regulation (EC) N° 1901/2006 (Paediatric Regulation), to incentivise the development of off-patent medicines exclusively for use in the paediatric population [2]. The PUMA concept was considered necessary as many medicines used in children have never been adequately tested and are often not available in child-appropriate dosage forms. A systematic review from 2018 showed that off-label drug use in children varies by country and care setting, with reported rates ranging from 3.2 to 95% [3]. Therefore, the main goal of the PUMA concept is to stimulate research to help transform known paediatric off-label use into authorised use that is supported by safety and efficacy evidence [2]. Disappointingly, by the end of 2022, only six products (Table 1) had been granted a PUMA, with few in the pipeline.

**Table 1:** Drugs authorised under paediatric use marketing authorisation

Drug	Brand	Formulation	Marketed strength/concentration	Year of authorisation in EU	Initial authorised indication
Midazolam	Buccolam®	Oromucosal solution	2.5 mg in 0.5 mL 5 mg in 1 mL 7.5 mg in 1.5 mL 10 mg in 2 mL	2011	Treatment of prolonged, acute, convulsive seizures in infants, toddlers, children and adolescents (from 3 months to < 18 years)
Propranolol	Hemangiol®	Oral solution	3.75 mg in 1 mL	2014	Treatment of proliferating infantile haemangioma requiring systemic therapy. It is to be initiated in infants aged 5 weeks to 5 months
Glycopyrronium	Sialanar®	Oral solution	320 µg in 1 mL	2016	Symptomatic treatment of severe sialorrhoea (chronic pathological drooling) in children and adolescents aged 3 years and older with chronic neurological disorders
Hydrocortisone	Alkindi®	Granules in capsules	0.5 mg 1 mg 2 mg 5 mg	2018	Replacement therapy of adrenal insufficiency in infants, children and adolescents (from birth to < 18 years old)
Melatonin	Slenyto®	Minitablets	1 mg 5 mg	2018	Treatment of insomnia in children and adolescents aged 2–18 with autism spectrum disorder and/or Smith-Magenis syndrome, where sleep hygiene measures have been insufficient
Vigabatrin	Kigabeq®	Soluble tablets	100 mg 500 mg	2018	Indicated in infants and children from 1 month to less than 7 years of age for monotherapy of infantile spasms (West’s syndrome); in combination with other antiepileptic medicinal products for patients with resistant partial epilepsy (focal onset seizures) with or without secondary generalisation, that is where all other appropriate medicinal product combinations have proved inadequate or have not been tolerated

The European Commission’s 10-year report on the impact of the Paediatric Regulation cited several concerns, including the continued off-label use of generic medicines containing the same active ingredient at lower costs, unfavourable pricing and reimbursement practices, as reasons for the low interest in PUMAs among medicines developers [2]. Recent observations have also highlighted disparities in the availability of these products across EU countries and inconsistent reimbursement policies, which further undermine the principle of the PUMA framework in ensuring equity in access to safe, effective and high-quality medicines [2, 4–7]. However, data on the accessibility of PUMA products at the healthcare provider level are limited. This study aimed to examine the availability of and access to PUMA products in the United Kingdom (UK) and to identify factors determining patient access to these products in clinical practice.

## Methods

We conducted a thematic analysis of a UK-based online discussion forum hosted by the Neonatal and Paediatric Pharmacy Group (NPPG). This forum, with approximately 500 members at the time of this study, serves as a platform for paediatric pharmacy professionals to discuss paediatric pharmaceutical care topics in both community and hospital settings. The analysis aimed to provide qualitative insights from these professionals regarding the use of PUMA products (Table 1) in clinical practice. Additionally, a web-based survey was distributed through the NPPG to investigate the availability of PUMA products across UK hospitals. It should be noted that the six PUMA products were authorised under the centralised procedure and granted marketing authorisation by the European Commission, which is valid in all EU Member States; the UK was an EU member state at the time these marketing authorisations were granted, and their authorisations were automatically converted to UK marketing authorisations on 1 January 2021.

### Data collection

All archived forum posts from 2011 to February 2022 were searched using the brand name of the six PUMA products and their generic names as search terms. A researcher (MW) who is a member of the NPPG discussion forum retrieved the relevant discussion threads, removed personal identifying information and imported them into NVivo software (version 11) for analysis. Additionally, a web-based survey was distributed via email to NPPG members in September 2022 for a 4-week period. The survey was designed using Qualtrics Survey Software and consisted predominantly of multiple-choice questions with an open text field to clarify the answer where required. The draft survey was reviewed by practising pharmacy professionals who provided feedback on the structure and clarity of the questions.

### Data analysis

Two researchers (MW and AH) conducted an in-depth analysis of the forum posts. All forum posts were read multiple times to ensure familiarisation with the content and subsequently screened to identify relevant discussion threads. Discussion threads that did not specifically address the PUMA products were excluded. The relevant discussion threads were independently coded line by line by the two researchers. Inductive thematic analysis was used to identify emerging themes which were refined through discussions until agreement is reached. The researchers also revisited all posts to ensure the themes captured all relevant comments, resulting in a comprehensive representation of the dataset.

The survey data were analysed quantitatively using descriptive statistics, with the results presented as number, percentage and mean (standard deviation (SD)) unless otherwise specified. Data on product availability were analysed at the hospital level to avoid duplication of responses from the same hospital for the same product. The free-text responses from the survey were analysed qualitatively using thematic analysis.

### Ethical considerations

The study was exempted from ethical review as forum posts were de-identified and analysed anonymously. Forum users were aware that their responses would be available for other members to view online (the researcher who was responsible for data extraction was a member of the NPPG), and therefore, informed consent was not sought from forum participants. The survey only collected the names of the respondents’ workplaces, and no sensitive personal information was collected. Completion of the survey was assumed as consent.

## Results

The search of the discussion forum identified 117 discussion threads. Of these, 88 threads were excluded as they did not specifically address the PUMA products (e.g. use of intravenous midazolam, use of unlicensed melatonin solution). The remaining 29 threads, containing a total of 157 posts, were screened as relevant and included in the analysis.

A total of 118 participants accessed the survey link with 71 (60%) completing at least the first topic-related question. Three participants were excluded as they represented hospitals located outside of the UK. The 68 participants had a mean work experience in the pharmacy profession of 11.1 years (SD = 8.4), and 73.5% (50/68) had worked in a paediatric pharmacy for more than 5 years. The participants represented a total of 47 hospitals (including 11 of the 14 largest children’s hospitals in the UK).

### Availability of PUMA products

**Fig. 1:**
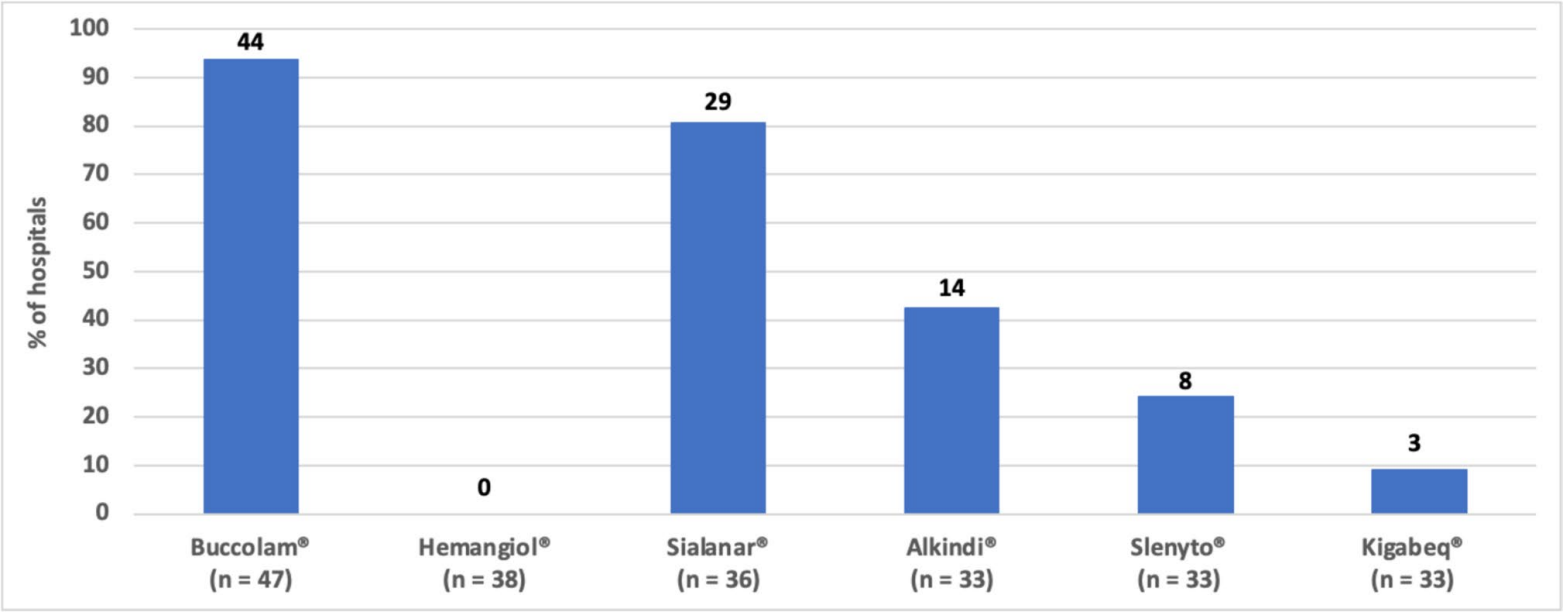
Availability of PUMA products. *n* = represents the number of hospitals that answered the survey question. The number above each bar indicates the number of hospitals that stocked the product

**Fig. 2:**
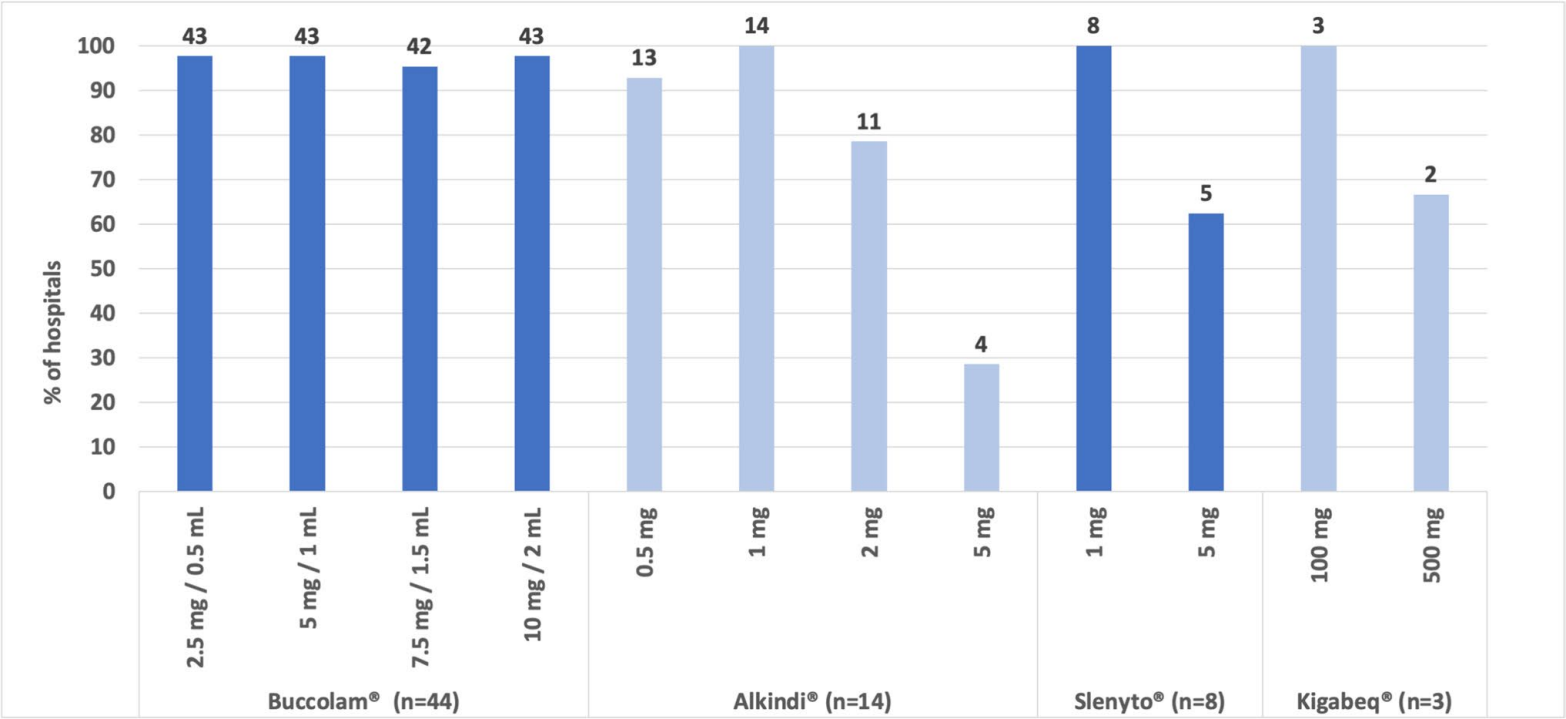
Availability of PUMA products by product strength (only PUMA products with multiple strengths are included). *n* = represents the number of hospitals that stocked at least one strength of the product. The number above each bar indicates the number of hospitals that stocked the product strength

Figure 1 shows the percentages of hospitals that stocked each of the six PUMA products. The availability of each PUMA product ranged from 0 to 93.6%, of which Buccolam^®^ oromucosal solution was most widely stocked (93.6%, 44/47). The percentages of hospitals that stocked Sialanar^®^ and Hemangiol^®^ solutions were 80.6% (29/36) and 0% (0/38), respectively. For Buccolam^®^ which is available in 4 strengths, 95.5% (42/44) of the hospitals stocked all the available strengths (Fig. 2). For Alkindi^®^, Slenyto^®^ and Kigabeq^®^, which are also available in multiple strengths, the results show that a greater percentage of hospitals stock the lower strengths compared to the higher strengths (Fig. 2).

### Thematic analyses

The qualitative findings are presented as themes with illustrative quotes in Tables 2 and 3.

**Table 2:** Themes (authorisation and availability) and subthemes identified from the discussion forum and survey

Theme	Subthemes	Illustrative quotes
Authorisation	Indications not covered by product marketing authorisation	Buccolam^®^ oromucosal solution—“The BNFc gives dosing information on buccal midazolam for conscious sedation, but these are weight-based and the resultant part-doses of a syringe are not very practical to administer” Slenyto^®^ melatonin prolonged release mini-tablets—“Use of licensed product for ASD, ASD + ADHD still under review in the community as most of our hospital patients will not fit these criteria”
	Age groups not covered by product marketing authorisation	Buccolam^®^ oromucosal solution—“The only disadvantage is that it not suitable for infants & < 3 months old on a microgram/kg dose”
Availability	Market availability	Hemangiol^®^ solution—“Although this product appears to be licensed in the UK, I don’t think it is actually available here”
	Unawareness of availability	Kigabeq^®^ soluble tablets—“Not aware that the product exists” Slenyto® melatonin prolonged release mini-tablets—“Not aware that the product exists”
	Lag time in local procurement/reimbursement decision	Alkindi^®^ hydrocortisone granules—“Awaiting formulary submission and approval process” Slenyto^®^ melatonin prolonged release mini-tablets—“New drug panel application pending”
	Slower procurement turnaround time of PUMA products relative to unlicensed products	Unlicensed melatonin solution—“Better procurement turnaround time”

**Table 3:** Themes (affordability, appropriateness, and acceptability) and subthemes identified from the discussion forum and survey

Theme	Subthemes	Illustrative quotes
Affordability	Higher cost of PUMA products relative to unlicensed products or off-label use of licensed products	Alkindi^®^ hydrocortisone granules—“We have decided to just keep the 0.5 mg and 1 mg strength. We are going to continue to use half or quarter a 10 mg tablet for other doses for cost saving reasons” Slenyto® melatonin prolonged release mini-tablets—“We have switched to Circadin (crushed if they can’t swallow) and the clinical commissioning group has agreed that primary care can prescribe this. Unlicensed liquid is second line if needed”
Appropriateness	Risk of medication errors	Sialanar^®^ solution—“Dosing in BNF is for 400mcg/ml glycopyrronium bromide however the more obvious strength on Sialanar packaging is 320 µg per ml so always causes confusion when prescribing and administering” Sialanar® solution—“strength confusion has reduced the acceptability of Sialanar in primary care”
	Practicality of medicine administration	Slenyto^®^ melatonin prolonged release mini-tablets—“Circadin first line but unlicensed liquid Ascomel as 2nd line for tube fed children who have difficulties with crushed tablets” Alkindi^®^ hydrocortisone granules—“Ease of administration—3.5 mg = 3.5 ml rather than 2 mg + 1 mg + 0.5 mg capsule”
Acceptability	Better patient acceptability with unlicensed products	Slenyto^®^ melatonin prolonged release mini-tablets—“The majority of the patients that are using Kidnaps are legacy patients who we are having trouble switching to circadin or slenyto or who are tube fed” Alkindi® hydrocortisone granules—“For children who do not accept Alkindi granules”

### Theme 1: authorisation

While PUMA products are authorised medicinal products, several posts about Buccolam^®^, Sialanar^®^and Slenyto^®^ indicated that details of the authorisation, particularly regarding age groups and therapeutic indications, are often perceived as insufficient to fully address clinical needs (illustrative quotes presented in Table 2). Participants commented on clinical needs outside of the approved conditions of use and reported the continued off-label use or use of unlicensed products in these situations. Notably, there were repeated comments on the use of unlicensed midazolam buccal solution for neonates and unlicensed melatonin solution for indications not covered by Slenyto^®^ marketing authorisation.

### Theme 2: availability

A medicinal product may receive authorisation but is not always marketed or made available for sale in a specific country. In this context, availability refers to the product being present on the market and commercially available for purchase by healthcare providers (illustrative quotes presented in Table 2). Forum members discussed the unavailability of Hemangiol^®^ solution in the UK, consistent with survey results indicating that it is not stocked by any of the surveyed hospitals. There was a subtheme of a lack of awareness of product availability, with some survey respondents reporting no knowledge of Slenyto^®^ and Kigabeq^®^ products, which received marketing authorisation in 2018. The lack of awareness of Kigabeq^®^ is reflected in the absence of forum discussion about this product. There was a further subtheme suggesting a significant lag between initial product marketing authorisation and local procurement/reimbursement decision.

### Theme 3: affordability

Affordability was the most common theme reported by forum members and survey respondents, particularly regarding Sialanar^®^, Alkindi^®^ and Slenyto^®^. Forum members indicated that, due to financial constraints, the higher cost of some of these products negatively influenced their hospital’s local procurement decisions (illustrative quotes presented in Table 3). The analysis showed that although Alkindi^®^ hydrocortisone granules are available in four strengths, only four hospitals (12.1%, 4/33) stocked all available strengths, with forum comments indicating continued use of unlicensed products as well as the off-label use of other hydrocortisone products that involves the manipulation of the dosage form. Similarly, the continued use of unlicensed melatonin solutions and off-label use of melatonin tablets were repeatedly reported by participants.

### Theme 4: appropriateness

The fourth theme relates to healthcare providers’ perspectives on the appropriateness of medicines, and this was noted for Sialanar^®^, Alkindi^®^ and Slenyto^®^. This theme is characterised by pharmacy professionals’ concerns about the potential risk of medication errors associated with product use and their reserved opinions on practical aspects of medicine administration (e.g. challenges faced by carers, difficulties with administration via enteral tubes). Consideration for these factors in influencing local procurement/reimbursement decisions was described, where continued off-label use and use of unlicensed products were considered more appropriate (illustrative quotes presented in Table 3).

### Theme 5: acceptability

Poor patient acceptability was reported for Alkindi^®^ and Slenyto^®^. Forum members and survey respondents reported patient preference for and continued use of unlicensed products or off-label use of licensed products in these situations (illustrative quotes presented in Table 3).

## Discussion

This study builds on previous efforts to evaluate the PUMA framework by examining patient access to PUMA products at the healthcare provider level. In this broader context, this study identifies various factors that hinder patient accessibility and shows a fragmented pattern of access across UK hospitals. It also highlights a concerning trend of continued off-label use and the use of unlicensed products, which contradicts the commitment to ensuring that children have access to medicines that have been appropriately researched and developed for the paediatric population.

The six PUMA products received centralised marketing authorisation; this means they are approved for use across the entire EU market (at the time, the UK was an EU member state). However, the availability of these products at the country level is not assured, as illustrated by Hemangiol^®^ which is not marketed in the UK. One possible reason for this marketing decision could be the developer’s concern regarding the continued off-label use of other authorised products available in the same dosage form (i.e. oral solution). Similarly, although Buccolam^®^ received marketing authorisation in 2011 and offered an innovative, easy-to-administer dosage form, it was marketed in only two-thirds of EU countries by 2020 [8]. The disparity in product availability across countries is not unique to PUMA products and is seen in other centrally approved medicinal products, including those with orphan designation, which, like PUMA products, operate in niche markets [9, 10]. Notably, small and medium-sized enterprises often play a crucial role in developing these products but generally encounter limitations in infrastructure and logistics, which can delay timely access across all EU markets and beyond. Additionally, it has been suggested that one of the primary market access challenges for the industry lies in national pricing and reimbursement policies [2, 11].

The issue of affordability remains a complex notion in healthcare, and this study did not set out to examine the clinical and economic evidence of these PUMA products. The pricing and reimbursement of medicinal products vary among countries and even sometimes within countries. In England, national clinical and cost-effectiveness assessment does not apply to the five marketed PUMA products, and therefore, the availability of these products for prescribing is determined by local decision-making. In this study, the observed variation in access, particularly in the case of Alkindi^®^ and Slyento^®^, could in part be attributed to divergent funding decisions reflecting differences in local approaches to prioritisation and resource allocation. Interestingly, national assessments are in place for these products in Scotland, which, by virtue, should facilitate equitable access in the region, although this study was not designed to test such a hypothesis. Nonetheless, it is worth noting that all five marketed PUMA products, with the exception of Slyento^®^, have received approval for use across NHS Scotland from the Scottish Medicines Consortium (health technology assessment agency for Scotland) and were deemed to have an acceptable budgetary impact compared to the standard of care [12–15].

A recurrent observation relating to the theme of affordability is the continued practice of dosage form manipulation and the use of unlicensed products. Forum members and survey respondents described the perceived higher price of PUMA products at face value relative to their current practice as a reason for continuing off-label use or use of unlicensed medicines. This conveys a concerning message as it not only undermines the goal of ensuring children have access to medicines that are appropriately authorised, but it also indicates continued professional support for a practice that increases the risk of dosing inaccuracy and inconsistency. Recognising the investment made by the industry and the unmet need for paediatric medicines, an alternative pricing and reimbursement framework that considers the budgetary constraints of healthcare providers while incentivising the industry to invest in the development of paediatric medicines should be a priority for policymakers. It is worth noting that these products pose unique challenges related to affordability, especially considering the ongoing availability of less expensive authorised products with established off-label use in children. In this regard, it is essential to expand awareness of the value of authorised age‐appropriate formulations.

Beyond affordability, a key theme that emerged from the present study is the view that these products do not address all known paediatric off-label use. The narrow scope of the approved conditions of use of these products (e.g. Buccolam^®^ is not approved for conscious sedation and Slenyto^®^ is not approved for the treatment of insomnia in children other than those with autism spectrum disorder and/or Smith-Magenis syndrome) is to be expected; initial marketing authorisations of medicinal products are almost always narrow as the industry aims to minimise the timeline to market entry for earlier investment returns. Nevertheless, the gap between approved conditions of use and remaining unmet clinical needs creates a situation where the prohibition of similar unlicensed products or off-label use, as advocated by the industry, cannot be practically regulated at the legislative level. The consequence is for healthcare providers to make decisions on which products to make available for which of their patients. While the narrow scope of the approved conditions of use of these products should, theoretically, not impact the reimbursement decisions for the approved conditions of use and there is some supporting evidence from several organisations [16, 17], it is not possible to draw firm conclusions that it did not influence local decision-making at all.

Lastly, the themes of appropriateness and acceptability also emerged as determinants influencing patient access to child-appropriate medicines. While PUMA products are developed exclusively for paediatric use with considerations for acceptability in children, the study findings suggest that the suitability of a product, as assessed by healthcare professionals and patients themselves, may not have been fully addressed by medicine developers. Nonetheless, it is well recognised that the pharmaceutical development of paediatric medicines can be challenging since this is a diverse patient population with specific needs. Greater collaboration between formulation experts and clinical colleagues, including healthcare professionals, in the drug development process is thus advocated.

There are some limitations to consider in the interpretation of the present findings. This study included a single country, which limits the generalisability of these findings to other health systems, although the pricing and reimbursement framework in the UK is generally considered more favourable. The incompleteness of some of the surveys is another limitation. In addition, the exact nature of the patient population of the hospitals surveyed may have influenced the need for a particular product, and thus whether holding stock was deemed necessary. Nonetheless, the survey respondents were from teaching hospitals and district general hospitals with paediatric departments that are likely to treat patients whose conditions fall within the authorised indications of the PUMA products.

## Conclusion

There exists limited data on accessibility to child-appropriate medicines. This study confirms the existence of inequality in patient access to PUMA products in the UK. By examining at the healthcare provider level, we identified determinants impeding patient access which can be categorised into five dimensions: authorisation, availability, affordability, appropriateness and acceptability.

The off-label use of medicines in children remains a common practice that requires attention. While policy measures aimed at stimulating investments in off-patent drug development in the paediatric setting are important, ensuring equitable access requires additional mechanisms to influence product availability within the health systems. A national approach to evaluating these products using health technology assessment methodologies can help reduce access inequalities and address the limitations of current local procurement practices, which often undervalue the critical attributes of safety, efficacy and quality endorsed by regulatory approval. Healthcare professionals must not be complacent about the risks associated with unlicensed and off-label use of medicines; continued education and training are essential to equip them with the skills needed to effectively communicate these risks and drive improvements in clinical practice and patient safety. More concerted actions are needed to better align the drug development framework, regulatory incentives, national policies, clinical and cost-effectiveness assessment and pricing and reimbursement decisions to bring these products into daily clinical practice.

## Data Availability

Data available on request from the authors.

## Declarations

### Disclaimer

The views expressed in this publication are those of the authors and not necessarily those of the NHS, the NIHR or the Department of Health and Social Care.
